# Cross-Sectional Study on Antibiotic Usage in Pigs in Germany

**DOI:** 10.1371/journal.pone.0119114

**Published:** 2015-03-18

**Authors:** Lisa van Rennings, Christiane von Münchhausen, Henry Ottilie, Maria Hartmann, Roswitha Merle, Walther Honscha, Annemarie Käsbohrer, Lothar Kreienbrock

**Affiliations:** 1 Department of Biometry, Epidemiology and Information Processing, WHO-Collaborating Centre for Research and Training in Veterinary Public Health, University of Veterinary Medicine Hannover, Hannover, Germany; 2 Institute of Pharmacology, Pharmacy and Toxicology, Veterinary Faculty, University of Leipzig, Leipzig, Germany; 3 Federal Institute for Risk Assessment, Berlin, Germany; INIAV, I.P.- National Institute of Agriculture and Veterinary Research, PORTUGAL

## Abstract

To be able to analyze the relationship between the level of resistance and the use of antimicrobials, it is necessary to collect detailed data on antimicrobial usage. For this reason, data on antimicrobial use on 495 pig farms from entire Germany were collected and analyzed. In Germany, each application and dispensing of medicines to food-producing animals is documented in detail obligatorily by the veterinarian. This information was collected retrospectively for the year 2011. The analyses undertook separate examinations on the age groups sow, piglet, weaner and fattening pig; both the route of administration and indication per active ingredient, and active ingredient class, were evaluated. In total, 20,374 kg of antimicrobial substances were used in the study population. Tetracyclines were used in highest amounts, followed by beta-lactams, trimethoprim-sulfonamides and macrolides. Concerning the frequency of using an active substance per animal, polypeptides were most commonly administered. In all age groups, respiratory infections were the main indication for using antimicrobials, followed by intestinal diseases in piglets, weaners and fattening pigs and diseases of reproductive organs in sows. Over a period of 100 days, the median number of treatment days with one antimicrobial substance for piglets was 15 days, for weaners about 6 days, for fattening pigs about 4 days and for sows about 1 day. A multifactorial ANOVA was conducted to investigate which factors are associated with the treatment frequency. The factors “veterinarian” and “age group” were related to the treatment frequency, just as the interaction between “veterinarian” and “farm size” as well as the interaction between “veterinarian” and “age group”.

## Introduction

The availability of antibiotics for treating bacterial diseases in animals must be preserved to ensure animal health and welfare. As any use of antimicrobial substances may be associated with the development of resistance [1], the wide distribution of antimicrobial resistance has become an increasing problem in human and veterinary medicine [2]. To limit further spread, actions were already taken in veterinary medicine. As an example, the use of antibiotics for growth promotion was banned in the European Union in 2006 (Regulation (EC) No. 1831/2003) [3].

To be able to assess the relationship between the observed resistance and the use of antimicrobials, it is necessary to provide detailed data on antimicrobial usage. In the Zoonoses Directive 2003/99/EC of the European Commission [4], the monitoring of antimicrobial resistance in zoonotic agents and other bacteria, which might be of relevance for public health in the Member States of the European Union is made mandatory. In contrast to this, up to now no legal requirements for the collection of data on consumption of antimicrobials has been implemented on EU level. Recently, European Medicines Agency (EMA) has published a reflection paper on how to collect data on consumption of antimicrobial agents in animals [5].

Years ago, active monitoring programs on antimicrobial consumption were established in some European countries, which have been continuously carried out to date (see e.g. [6–9]). In all of these monitoring systems data on antibiotic consumption are collected from pharmacies, wholesalers, and the pharmaceutical industry. However, these approaches only provide valuable data if prescription of the drugs to the different livestock populations is recorded. In Germany, veterinarians have the right to dispense. German veterinarians are allowed to obtain drugs by wholesalers and pharmaceutical companies, to deliver drugs to the animal owners and to produce and keep drugs. In addition, for each treatment in animals the veterinarian has to take the responsibility for. This has to be documented formally by means of the German pharmaceutical law [10]. As the regulations regarding the sale and delivery of antimicrobial drugs in Germany basically differ from those of other EU Member States, the concepts already established there cannot be applied for Germany due to the non-central federal state system and the right of veterinarians to sell drugs. Therefore, it is necessary to develop new concepts for appropriate data collection in Germany. Thus, developing a bottom-up approach for the detection of antimicrobials in animals was one of the goals formulated several times in the German Antibiotics Resistance Strategy [11]. This should be an important contribution to public health.

For this reason a feasibility study was conducted successfully in the years 2007 and 2008 and a method for collecting data on consumption of antimicrobials in farm animals was developed [12,13]. The aim of this recent study was to run a follow-up cross-sectional investigation. Emphasis was laid on collecting data from a representative subset of the major animal livestock populations to achieve a representative picture on antimicrobial usage in Germany.

## Material and Methods

### 1. Study Design

The study was set up as a cross-sectional study, based on voluntary participation of veterinarians and farmers. To ensure the representativeness for the whole of Germany, an appropriate sampling plan was created. In a first step, Germany was classified in four regions: Northwest, Middle, East and South, already defined by Merle et al. [14]. These regions were defined on the basis of their agricultural structures. Region “East” is marked by relatively few huge farms. In contrast, region “South” is characterized by many small farms. In region “Northwest”, there is a high density of livestock, in region “Middle” only a moderate density of livestock is present. In a second step, districts, which are typical for these regions, were identified with regard to an average number of livestock in the region. From these districts participants were recruited. All participants were informed of the study's aim and gave written consent for data to be used for research purposes. The study is addressing data, which were taken from the forms, on which treatments of animals and the delivery of animal drugs to animal owners are documented by the veterinarian. With these forms each application and dispensing of medicines to food-producing animals by the veterinarian is documented obligatorily in Germany. Our study based on official forms, on which any treatment administered to the animal is documented by the veterinarian. Because these documents have to be filled in obligatory, naturally no illegal treatments are noted on these. It should be stressed here in addition that prophylactic usage of antibiotics is not allowed in Germany. German veterinarians adhere to special guidelines on the prudent use of antibiotics in veterinary medicine [1,15].

All participants gave consent to use the data for this study, given that all data of pig holding farmer and veterinarian were pseudonymized before the data were analyzed. This was stated in a privacy statement with each participant. The study protocol was approved by the sponsor (the Federal Institute of Risk Assessment) and announced to the data protection officers of both universities.

The recruitment of participants and input of their data was carried out in the period from May 2012 until February 2013. Data were collected retrospectively for the calendar year 2011.

### 2. Data collection and analysis

The data collection was implemented following the concepts from the feasibility study in 2007/2008 [13]. Data were taken from the forms, on which treatments of animals and the delivery of animal drugs to animal owners are documented by the veterinarian. With these forms each application and dispensing of medicines to food-producing animals by the veterinarian is documented obligatorily in Germany. Basic information of these documents are treatment or delivery date, the name of veterinarian and farmer, number, type and identity of the animals to be treated, diagnosis, drug name, amount of applied or submitted drug, charge number, dosage per kg per day as well as type and duration of administration and the withdrawal period.

In addition, an animal-specific questionnaire was sent to participating farmers, who should give information about farm management, keeping of animals, and also the number of animals kept as well as livestock places.

The data were entered manually into a database developed during the feasibility study—here the Open-Source relational database system MySQL was used in its current version (currently 5.0. 51a). There were mandatory fields defined in the input mask, which complied with the mandatory information on the treatment and delivery form. To enable fast manual entering of data, drop-down lists were applied. Diagnosis was linked to eight predetermined diagnostic groups (udder disease, skin disease, respiratory disease, intestinal disease, central nervous system disease, joint disease, reproductive organs and other diseases). One entered data record corresponded to one treatment. All data records were checked for completeness and pharmacological plausibility, both during data entry as well as during a final extensive plausibility checkup, taking into account information stated in the additional farmer's questionnaire received.

In the context of the analysis, each age group, defined by weight and production system was considered separately. Thus, the four age groups piglet (4 kg on average), weaner (15 kg on average), fattening pig (50 kg on average) and sow (200 kg on average) were distinguished. Allocation to these groups was done to the best extent possible on the basis of the information documented on the identity of the animals treated or the type of pigs kept on the farm. It was assumed that a sow is housed for more than one year, so the time period under risk of a sow was stated as one year. A piglet is suckled 28 days on average before it is transferred to the next production phase. In Germany the average production phase of a weaner is about 46 days. With a service period of about seven days between groups, this yields approximately 6.89 passages for weaning units per farm per year. In Germany, a fattening pig is fattened 115 days on average. With a subsequent time-window for cleaning and disinfection of 14 days, 2.83 fattening passages arise per year and per farm on average [16]. Considering these average values the reporting of the antimicrobial use within the different age groups refers to one production period.

In the context of the analysis, the participating pig farms were divided into various farm size groups based on the numbers of livestock places per farm. To achieve this, the farm size groups “small”, “medium” and “large” were defined for each age group on the basis of tertiles derived from our data. Medium-sized farms were defined as those with 80 to 199 sows, 820 to 2,049 piglets, 330 to 799 weaners and 370 to 999 fattening pigs.

The analysis involved the determining of the used active ingredients included in the drug applied and active ingredient quantities per age group. Focus of the analysis was laid on the calculation of amounts of antimicrobial substances and as well on the calculation of number of treatments per active ingredient and animal (treatment unit or number of used daily doses (nUDD)), and the calculation of treatment days per active ingredient per animal (treatment frequency). A treatment unit (UDD) is equivalent to the administration of one active ingredient to one animal on one day [13,17]. Based on this, the sum of all treatment units (nUDDs) in an observed population over an observation period can be calculated as follows:

nUDD(numberofuseddailydoses)=numberoftreatedanimals×numberoftreatmentdays×numberofactiveingredients

The treatment frequency specifies how many days an animal in a herd is treated with one active ingredient on average [13,17]. The treatment frequency (TF) is calculated using:

$$TF\;=\frac{nUDD}{population\;size}$$

To calculate the treatment frequency for each farm per year, the number of animals kept per farm per year had to be determined. For this purpose, the livestock places per farm and age group were multiplied by the average number of production periods per farm per year (sow = 1; weaner = 6.89; fattening pig = 2.83). The number of piglets per farm and year was always estimated for any one farm keeping sows, by multiplying the number of sows on this farm by the average number of litters per sow and year (2.35) and the average number of weaned piglets per sow and litter (10.25).

In the course of the analysis, also factors were analyzed which were assumed to have an influence on the treatment frequency. These factors were analyzed for each age group separately via a multifactorial analysis of variance (ANOVA, target variable: logarithmized treatment frequency, standardized per 100 days of animals kept). Region and farm size were examined as fixed factors; the responsible veterinarian was examined as random factor. For model selection backward selection procedures were used.

Statistical analyses were performed using SAS, version 9.3 TS level 1M2 (SAS Institute Inc., Cary, NC, United States).

## Results

### 1. Study Population

In total, 18,151 data records of 495 pig farms from entire Germany were collected and analyzed. On the 495 participating pig farms, one or several age groups were kept, giving a total of 945 groups (sow, piglet, weaner, fattening pig). These groups were allocated as follows: 215 farms keeping sows, 199 farms keeping piglets, 187 farms keeping weaners, as well as 344 farms keeping fattening pigs. In relation to the entire German pork production the study covered 2.19% of all livestock places for sows, 6.14% for piglets, 3.17% for weaners and 2.85% for fattening pigs in Germany (data source from Federal Statistical Office [18]).

The analysis of the data was carried out in two different ways. On the one hand, the amount of antimicrobial agents consumed was determined separately for the different age groups. This was reported with consideration of the season, the administration route and the indication. On the other hand, the frequency of treatments was analyzed separately according to age group.

### 2. Used quantities

In total, 20,373.6 kg of antimicrobial agents were used on the study farms in 2011. Tetracyclines were most commonly used with a consumption quantity of 7,275.5 kg (35.7%), followed by beta-lactams with 6,720.9 kg (33.0%), potentiated sulfonamides (trimethoprim-sulfonamides) with 2,126.6 kg (10.4%) and macrolides with 2,827.7 kg (13.9%). Polypeptides (858.4 kg, 4.2%) and aminoglycosides (248.2 kg, 1.2%) were in the fifth and sixth position regarding consumption quantities. The consumption quantities per active ingredient were also evaluated separately for each age group. In addition, seasonal variations in the consumption of certain antimicrobial substances were examined according to the age group (Table 1).

**Table 1:** Seasonal effects regarding consumed quantities taking the example of selected used active ingredients in highest amounts.

active ingredient	Consumed quantities in kg				
	spring	summer	autumn	winter	total
	**sow**				
**Amoxicillin**	66.9	75.4	23.6	54.7	220.5
**Chlortetracycline**	23.2	27.5	26.1	21.0	97.9
**Colistin**	2.4	1.4	1.5	1.0	6.3
**Sulfadiazine**	237.6	97.2	295.2	216.0	846.0
**Tetracycline**	592.3	134.9	100.0	118.3	945.5
**Tylosin**	2.6	11.3	3.6	1.0	18.5
	**piglet**				
**Amoxicillin**	304.8	446.8	452.4	200.5	1,404.6
**Chlortetracycline**	38.4	36.2	62.9	16.7	154.2
**Colistin**	40.9	68.7	60.7	36.9	207.2
**Sulfadiazine**	113.4	75.8	132.1	56.9	378.2
**Tetracycline**	116.7	180.2	220.6	133.5	651.0
**Tylosin**	40.3	21.9	19.3	21.7	103.1
	**Weaner**				
**Amoxicillin**	907.2	480.9	547.8	753.6	2,689.5
**Chlortetracycline**	228.0	182.0	108.1	129.6	647.7
**Colistin**	164.4	136.0	77.5	151.1	528.9
**Sulfadiazine**	153.6	134.4	111.4	187.6	587.0
**Tetracycline**	385.2	268.4	304.2	334.0	1,291.9
**Tylosin**	36.9	34.8	26.4	35.3	133.4
	**fattening pig**				
**Amoxicillin**	574.2	505.8	535.9	652.7	2,268.6
**Chlortetracycline**	361.1	327.0	326.3	413.5	1,427.8
**Colistin**	42.0	21.3	26.7	25.8	115.9
**Sulfadiazine**	91.4	107.4	173.5	104.8	477.2
**Tetracycline**	302.2	364.5	199.9	240.1	1,106.6
**Tylosin**	483.8	485.4	474.6	383.8	1,827.7

Among the sows, the active ingredients of trimethoprim-sulfonamides, tetracycline and amoxicillin were administered most. Tetracycline was used the most in spring. The consumption amount of tetracycline in the spring was about five-fold higher than the amount consumed in the other seasons. The highest amounts of trimethoprim-sulfonamides were consumed in autumn, and almost as much in summer. Amoxicillin was used in summer in highest amounts, but the least in autumn. The highest drug use for all active ingredients was detected in spring.

Among piglets, amoxicillin, tetracycline, trimethoprim-sulfonamides and colistin were used in highest amounts, also with seasonal differences. In contrast to the fattening pigs, amoxicillin was least consumed in winter, with a steady increase in consumption amounts from spring to autumn. Amoxicillin was used more than twice as much in autumn than it was in winter. This observation was also made for tetracycline, but with the lowest consumption level being in spring. For all active ingredients, the highest drug use in piglets was detected in autumn.

Among weaners, amoxicillin, tetracycline, chlortetracycline, and about equal proportions of trimethoprim-sulfonamides and colistin were used in highest amounts. In this age group, seasonal differences in regard to the consumed quantities of several active ingredients were also observed. Amoxicillin was consumed the most in spring, twice as much as in summer, with a steady rise in the consumption quantities from summer to spring. Distributed over the year, tetracycline as well as trimethoprim-sulfonamides were consumed constantly. However, the amounts of chlortetracycline were much higher in spring than in the second half of the year. In weaners, colistin was used the least in autumn, but rather constantly over the rest of the year. For all active ingredients, the highest drug use in weaners was detected in spring.

Amoxicillin, tetracycline, tylosin and chlortetracycline were used in highest amounts in fattening pigs and there were different seasonal patterns. Amoxicillin was more often used in winter than in the summer months, whereas the active ingredient tylosin was used about 100 kg less in winter than in the summer. For all active ingredients, the highest drug use for fattening pigs was detected from June to August, the lowest use in autumn.

From all 20,373.6 kg antimicrobial substances used, 19,903 kg (98%) were administered orally, and only 472.3 kg (2%) were administered per injection. However, looking at the data records, there were more than twice as many records on parenteral administration than on oral administration. Among the four age groups, especially for sows there were mainly records on treatment per injection. Regarding the fattening pigs, records on parenteral and oral treatments were approximately the same, with slightly more records on parenteral treatments. In contrast, for weaners records on oral administration were about twice as frequently as records on parenteral treatments. Among the piglets, there were more records on parenteral treatments than for oral administration.

In the course of the analysis, also the consumption amounts of antibiotic active substances according to several indications were determined for the different age groups (Table 2).

**Table 2:** Amounts of active ingredients per age group and indication group (selected active ingredients).

active ingredient	Consumed quantities in kg							
	respiratory disease	intestinal disease	joint disease	skin disease	other diseases	central nervous system	reproduc-tive organs	total
	**sow**							
**Amoxicillin**	125.8	1.0	8.5	0.8	73.8	-	10.6	220.5
**Chlortetracycline**	41.1	-	-	<0.1	21.5	-	35.3	97.9
**Colistin**	0.5	5.7	-	-	0.1	-	<0.1	6.3
**Sulfadiazine**	39.6	-	-	-	768.0	-	38.4	846.0
**Tetracycline**	776.2	4.6	-	-	89.6	-	75.1	945.5
**Tylosin**	1.1	12.5	0.9	-	2.7	<0.1	1.3	18.5
	**piglet**							
**Amoxicillin**	849.1	12.2	13.9	14.5	514.9	-	-	1404.6
**Chlortetracycline**	149.3	4.0	-	-	0.9	-	-	154.2
**Colistin**	2.1	200.4	-	-	4.6	-	-	207.2
**Sulfadiazine**	281.3	74.3	-	4.8	17.80	-	-	378.2
**Tetracycline**	571.5	-	-	-	79.5	-	-	651.0
**Tylosin**	6.8	88.1	-	-	8.3	-	-	103.1
	**weaner**							
**Amoxicillin**	2195.3	52.5	1.3	37.1	403.3	-	-	2689.5
**Chlortetracycline**	635.9	1.4	-	-	10.4	-	-	647.7
**Colistin**	5.3	520.4	-	-	3.2	-	-	528.9
**Sulfadiazine**	518.2	50.4	-	6.4	12.0	-	-	587.0
**Tetracycline**	1068.1	127.3	-	-	96.6	-	-	1291.9
**Tylosin**	5.1	112.2	-	-	16.1	-	-	133.4
	**fattening pig**							
**Amoxicillin**	1924.0	92.7	14.1	6.3	231.5	-	-	2268.6
**Chlortetracycline**	1175.7	181.6	-	2.8	67.7	-	-	1427.8
**Colistin**	6.4	95.2	-	-	14.5	-	-	116.1
**Sulfadiazine**	413.9	26.0	-	-	37.2	-	-	477.2
**Tetracycline**	830.9	103.3	-	2.8	169.6	-	-	1106.6
**Tylosin**	39.9	1747.3	0.1	-	46.1	-	-	1833.4

In piglets, the highest amounts of antimicrobial substances were used against respiratory diseases. In second and third place, "other diseases" and "intestinal diseases" were documented as reasons for treatment. Respiratory diseases in piglets were first and foremost treated with amoxicillin, but also with tetracycline. Intestinal diseases in piglets were mostly treated with colistin and tylosin.

Also in sows, the highest quantities of antimicrobial active ingredients were used against respiratory diseases. “Other diseases” were in second place, too, but in third place were diseases of the reproductive organs. Respiratory diseases in sows were particularly treated with tetracycline but also with amoxicillin. Diseases of the reproductive organs were mainly treated with amounts of tetracycline.

In fattening pigs and weaners, the highest quantities of antimicrobial substances were also used against respiratory diseases.

As in piglets, respiratory diseases in weaners were mainly treated with amoxicillin, but also with tetracycline. Intestinal diseases were especially treated with colistin, but also tetracycline and tylosin were used in approximately equal amounts.

Also in fattening pigs respiratory diseases were mainly treated with amoxicillin. In addition to amoxicillin, especially chlortetracycline played an important role in the treatment of respiratory diseases. The treatment of intestinal diseases was primarily carried out with tylosin.

### 3. Treatment units

**Fig 1:**
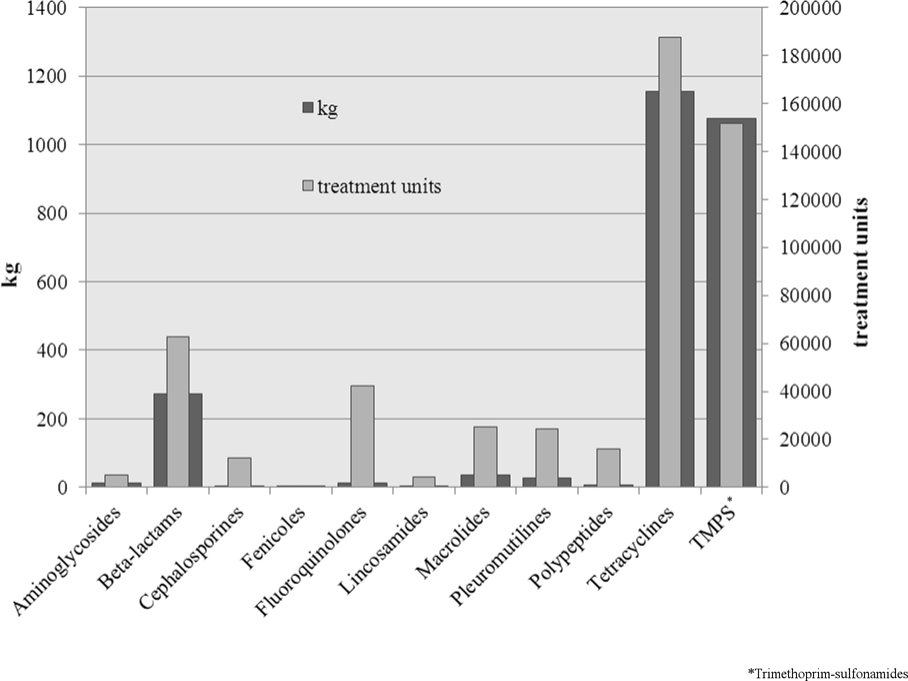
Used quantities and treatment units of applied classes of active ingredients for sows.

**Fig 2:**
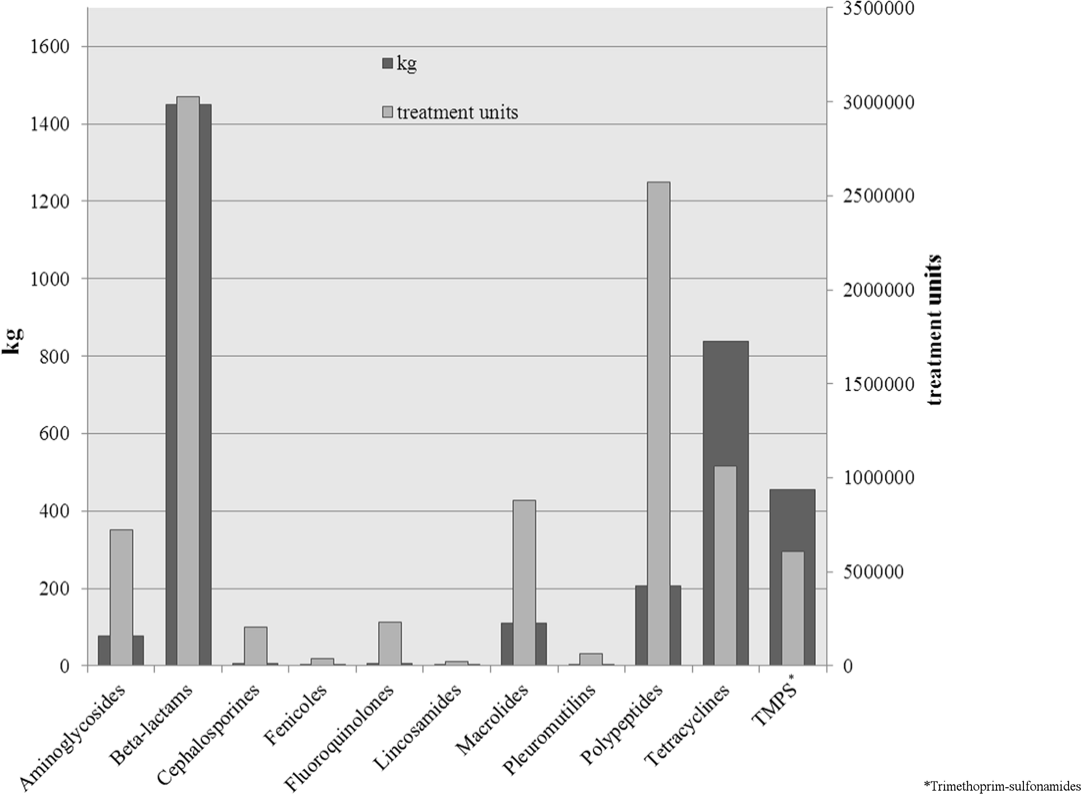
Used quantities and treatment units of applied classes of active ingredients for piglets.

**Fig 3:**
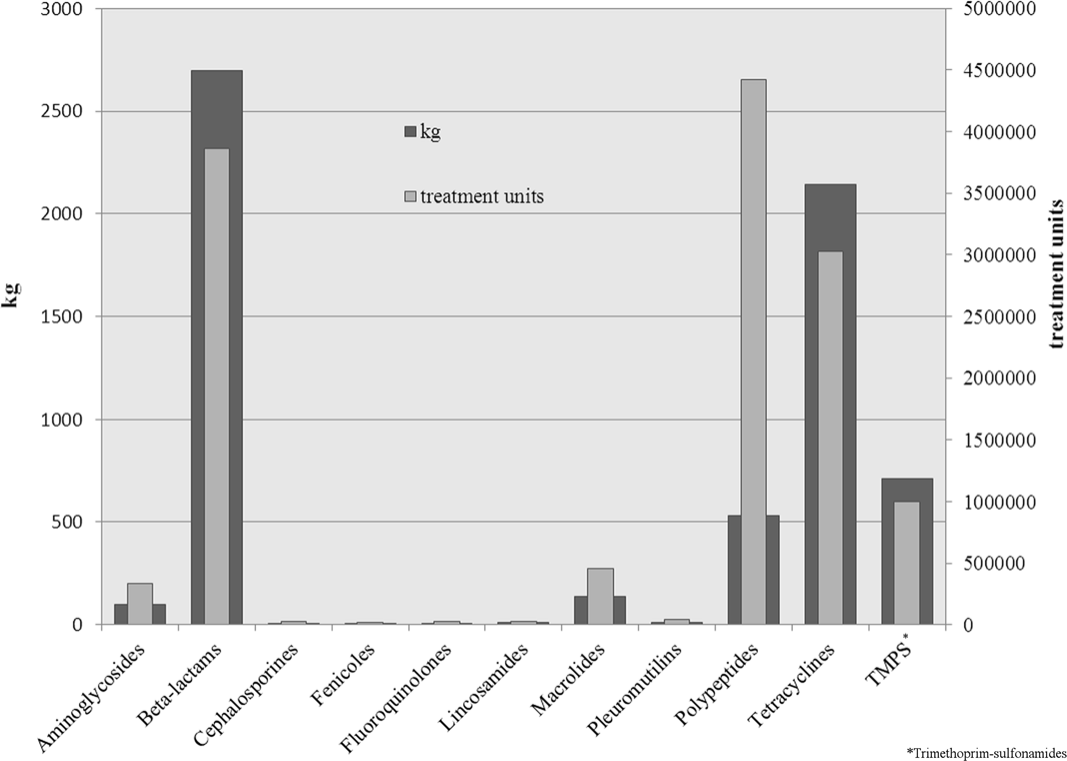
Used quantities and treatment units of applied classes of active ingredients for weaners.

**Fig 4:**
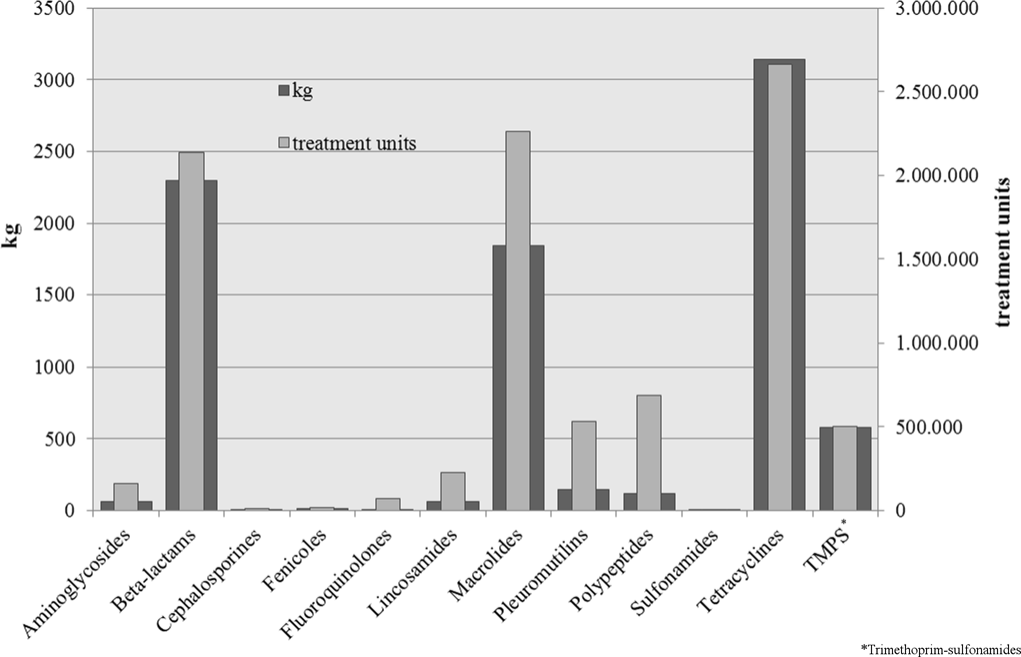
Used quantities and treatment units of applied classes of active ingredients for fattening pigs.

The amount of drugs applied is strongly influenced by the recommended dosage as well as the body weight of the treated animal group. Therefore, tons of used drugs may only give a rough picture of the situation. Thus, treatment units were calculated. To illustrate the difference between these two ways of analyses, the Figs. 1–4 show the used treatment units compared to the used quantities per age group. The great difference between the consumed quantity and the number of treatment units of some active ingredient classes is due to the different dosages of the active ingredients.

In sows, tetracyclines, trimethoprim-sulfonamides and beta-lactams are up front in terms of treatment units as well as the consumed quantities. It is remarkable that fluoroquinolones, which were only used in small quantities, are in fourth place, concerning the treatment units. Also macrolides, polypeptides and pleuromutilins take a relatively large proportion of the treatments in this age group.

In piglets, beta-lactams, tetracyclines and trimethoprim-sulfonamides were most commonly used in terms of quantity. Regarding the treatment units, polypeptides became more prominently used in contrast to the use of tetracyclines. Macrolides, which only are in fifth place in terms of quantity, were nearly used as often as tetracyclines. Only small quantities of aminoglycosides, fluoroquinolones and cephalosporins were used, but they accounted for a relatively large proportion of treatment units.

Among weaners, there are clear differences between consumed quantities and treatment units of each active ingredient class. In terms of quantity, especially beta-lactams, tetracyclines and trimethoprim-sulfonamides were used. Nonetheless, looking at treatment units, polypeptides were the most used antimicrobial class in this age group, followed by beta-lactams and tetracyclines.

In fattening pigs, tetracyclines were most commonly used with regard to the consumed quantities, followed by beta-lactams and macrolides. On the treatment unit scale, macrolides were ahead of beta-lactams. When directly comparing the consumed quantities and treatment unit scale, the total amount of used polypeptides was comparatively low, but the number of treatment units was higher. It becomes obvious that polypeptides and pleuromutilins are much more important as is shown when looking at the consumed quantities only.

### 4. Treatment frequencies

Looking at the treatment frequencies depicted as distribution of the farm specific treatment frequencies with regard to one year and one passage, respectively, it was calculated that a sow on a typical farm was treated with one active ingredient on 3.2 days (median) per year on average. A fattening pig was treated with one active ingredient on 4.2 (median) days in its 115-day fattening period, with a combination of two active ingredients on 2.1 days. In its 46-day production period, a weaner was treated with one active ingredient on 3.1 (median) days, with a combination of two active substances on about 1.5 days. Within its four-week suckling period, a piglet received treatment with one active ingredient on 4.1 (median) days.

In the following, the treatment frequencies with one active ingredient per farm and 100 days, given as median value of all farms, are described, to ensure the comparability between the age groups. Converted to 100 days, a median treatment frequency of 0.86 days arose for sows, 14.74 days for piglets, 6.62 days for weaners and 3.67 days for fattening pigs.

**Fig 5:**
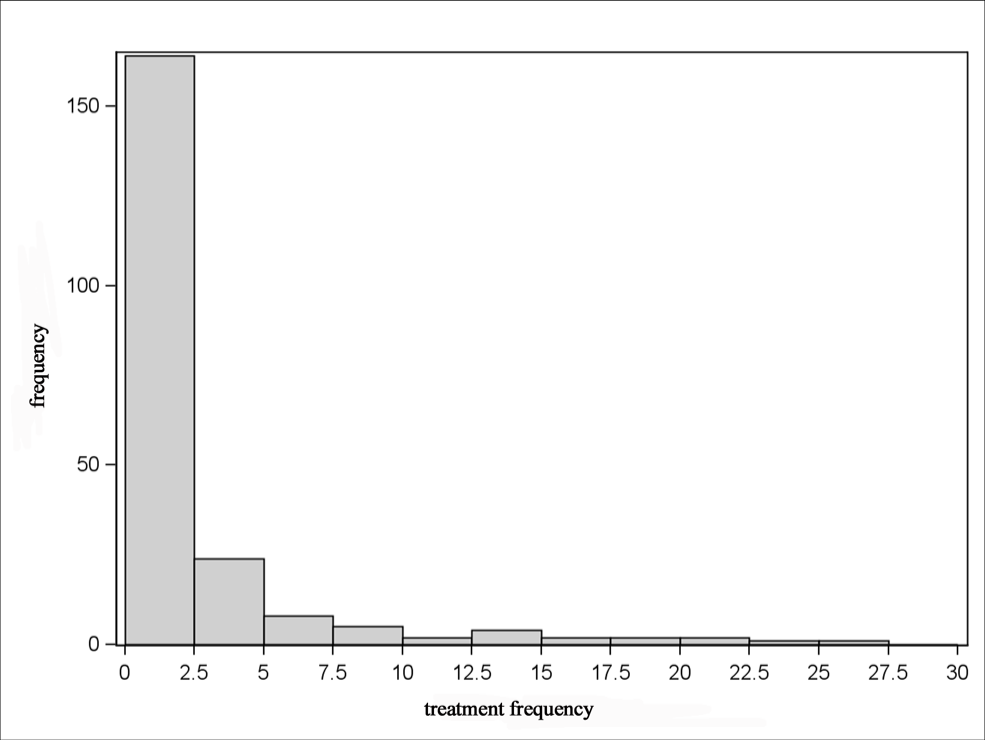
Distribution of treatment frequencies of sows.

**Fig 6:**
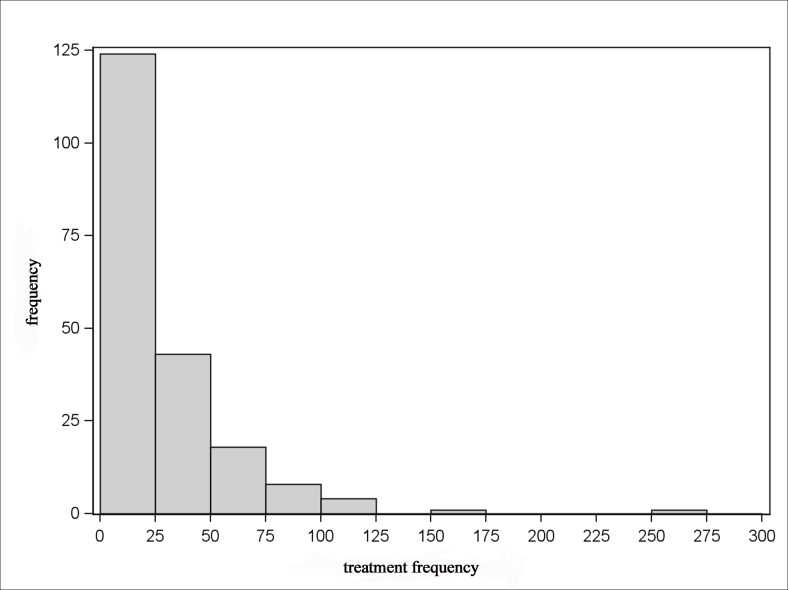
Distribution of treatment frequencies of piglets.

**Fig 7:**
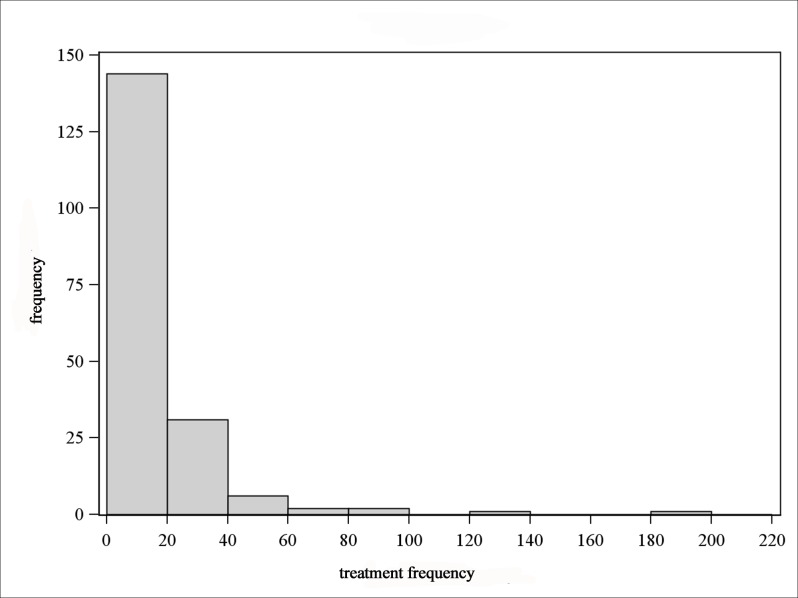
Distribution of treatment frequencies of weaners.

**Fig 8:**
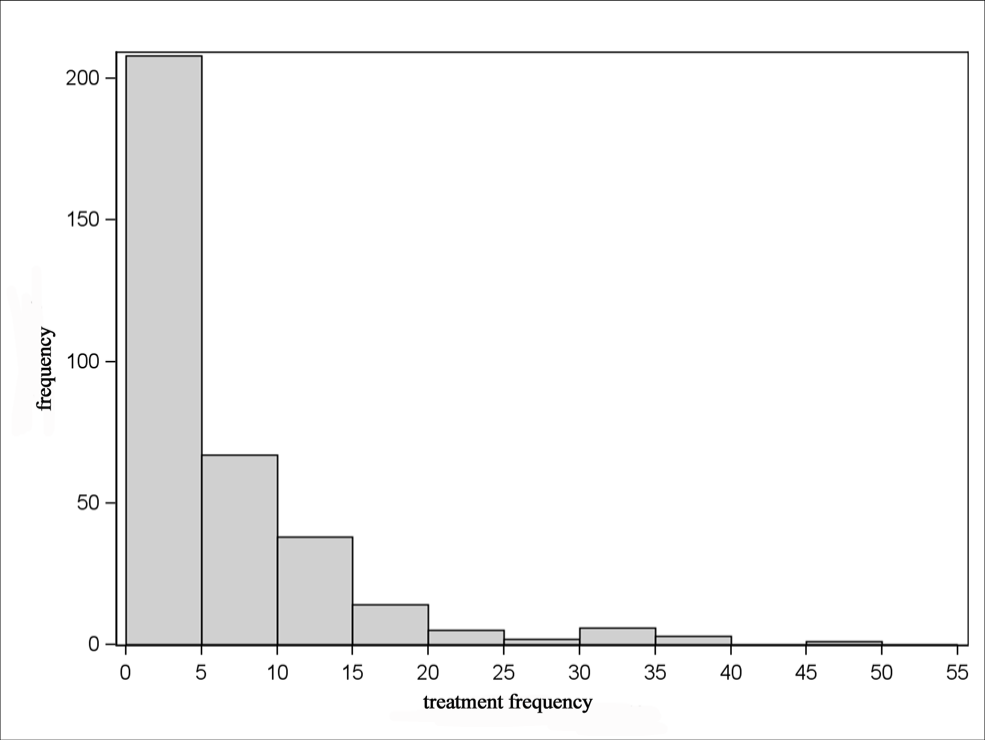
Distribution of treatment frequencies of fattening pigs.

The Figs. 5–8 show that the treatment frequencies across the farms are skewed to the right, in all age groups without exception. Therefore, the distribution is described with robust statistical measures only (Table 3). The comparison of all age groups shows that piglets were treated the most frequent and sows were treated the least.

**Table 3:** Distribution of the 100-day-treatment frequency per farm for the four different age groups.

age group	treatment frequency per 100 days							
	n	min	P5	P25	median	P75	P95	max
**sow**	215	0.005	0.023	0.26	0.86	2.39	13.32	26.43
**piglet**	199	0.006	0.593	4.78	14.74	35.71	93.65	261.86
**weaner**	187	0.011	0.126	2.20	6.62	17.75	45.50	180.80
**fattening pig**	344	0	0.031	0.92	3.67	7.84	19.09	45.62

In the course of the analysis, standardized therapy frequencies of individual active ingredient classes were calculated for the different age groups (Table 4). Within all age groups, beta-lactams represent the active ingredient class that was used on most farms. Thus, beta-lactams were used for treating sows on 167 farms (78% of all sow keeping farms), for treating piglets on 170 farms (85% of all piglet keeping farms), for treating weaners on 135 farms (92% of all weaner keeping farms) and on 260 farms for treating fattening pigs (76% of all farms keeping fattening pigs). Therefore, over a period of 100 days, a piglet was treated at about 4 days with beta-lactams (within the farms in which beta-lactams were applied). Furthermore, beta-lactams were used in sows at not even one day (0.2), weaners at about two days and fattening pigs at about one day (0.8).

**Table 4:** Distribution of the 100-day-treatment frequency per farm and active ingredient class for the four different age groups.

active ingredient class	Treatment frequency per 100 days							
	n	min	P5	P25	median	P75	P95	max
	**sow**							
**Beta-lactams**	167	0.003	0.020	0.05	0.17	0.51	1.69	12.47
**Aminoglycosides**	65	0.001	0.005	0.02	0.06	0.26	1.08	1.62
**Cephalosporins**	71	0.003	0.005	0.02	0.04	0.11	0.51	1.38
**Tetracyclines**	81	0.002	0.004	0.10	0.39	1.89	8.32	9.55
**Polypeptides**	7	0.064	0.064	0.95	2.23	7.56	11.34	11.34
**Fluoroquinolones**	111	0.002	0.011	0.04	0.15	0.42	1.28	3.17
**Lincosamides**	21	0.003	0.007	0.02	0.07	0.13	0.46	1.47
**Pleuromutilins**	12	0.003	0.003	0.07	0.26	0.78	3.65	3.65
**Makrolides**	55	0.002	0.002	0.02	0.05	0.19	1.44	10.58
**Trimethoprim-sulfonamides**	77	0.003	0.020	0.09	0.26	1.87	17.06	21.19
**Fenicoles**	17	0.002	0.002	0.01	0.02	0.04	0.27	0.27
**Sulfonamides**	.	.	.	.	.	.	.	.
	**piglet**							
**Beta-lactams**	170	0.006	0.153	1.11	4.33	10.06	28.86	58.97
**Aminoglycosides**	103	0.011	0.067	0.33	1.15	3.21	8.81	28.05
**Cephalosporins**	44	0.015	0.045	0.18	0.56	2.30	6.03	11.86
**Tetracyclines**	107	0.010	0.114	1.35	3.46	8.82	25.94	49.92
**Polypeptides**	110	0.126	0.432	3.50	9.73	19.96	48.24	98.59
**Fluoroquinolones**	120	0.033	0.070	0.27	0.56	1.51	4.39	14.01
**Lincosamides**	12	0.035	0.035	0.05	0.15	0.92	7.90	7.90
**Pleuromutilins**	8	0.185	0.185	1.46	2.72	4.12	31.95	31.95
**Makrolides**	97	0.013	0.064	0.64	1.42	4.45	31.12	76.22
**Trimethoprim-sulfonamides**	34	0.889	1.556	2.37	6.35	10.56	68.06	78.48
**Fenicoles**	16	0.019	0.019	0.16	0.75	2.08	12.72	12.72
**Sulfonamides**	.	.	.	.	.	.	.	.
	**weaner**							
**Beta-lactams**	135	0.003	0.081	0.74	2.20	5.44	19.42	64.68
**Aminoglycosides**	46	0.002	0.005	0.03	0.12	0.41	6.31	25.15
**Cephalosporins**	41	0.006	0.008	0.03	0.08	0.19	0.38	2.83
**Tetracyclines**	106	0.001	0.090	0.97	3.20	6.25	16.04	45.04
**Polypeptides**	103	0.011	0.463	2.07	5.14	9.26	21.39	68.66
**Fluoroquinolones**	60	0.003	0.008	0.03	0.10	0.26	1.52	2.63
**Lincosamides**	17	0.013	0.013	0.16	0.41	1.16	4.58	4.58
**Pleuromutilins**	10	0.009	0.009	0.44	1.35	2.06	3.13	3.13
**Makrolides**	73	0.004	0.009	0.08	0.31	1.36	14.16	23.67
**Trimethoprim-sulfonamides**	41	0.024	0.121	1.89	5.00	7.33	15.43	28.38
**Fenicoles**	17	0.000	0.000	0.03	0.08	0.25	1.57	1.57
**Sulfonamides**	.	.	.	.	.	.	.	.
	**fattening pig**							
**Beta-lactams**	260	0.004	0.009	0.07	0.81	3.09	8.74	16.88
**Aminoglycosides**	92	0.001	0.004	0.02	0.09	0.46	2.15	8.00
**Cephalosporins**	45	0.001	0.003	0.01	0.03	0.05	0.43	1.16
**Tetracyclines**	191	0.001	0.051	1.00	2.16	4.21	7.67	17.72
**Polypeptides**	62	0.004	0.161	0.66	1.78	4.06	12.90	16.57
**Fluoroquinolones**	154	0.003	0.008	0.03	0.07	0.18	0.68	8.75
**Lincosamides**	77	0.003	0.011	0.05	0.17	1.08	2.15	2.92
**Pleuromutilins**	45	0.002	0.006	0.46	1.13	2.39	5.44	11.67
**Makrolides**	159	0.002	0.005	0.04	0.25	1.52	5.30	25.48
**Trimethoprim-sulfonamides**	49	0.009	0.021	0.54	1.94	4.27	7.56	11.76
**Fenicoles**	57	0.001	0.005	0.02	0.04	0.12	0.77	3.80
**Sulfonamides**	1	.	.	.	0.71	.	.	.

As another example, polypeptides, which were used on 110 piglet keeping farms (55% of all farms keeping piglets) for treating piglets, were applied at about 10 days on these farms over a period of 100 days. In contrast, polypeptides were only used on 7 sow keeping farms (3% of all sow keeping farms). Within these farms, polypeptides were applied at 2.2 days to sows, but all in all they were not important for treating sows. Furthermore, polypeptides were used in 55% of all weaner keeping farms but only on 18% of all farms keeping fattening pigs. Within these farms, weaners were treated at about 5 days with polypeptides and fattening pigs at 1.8 days over a period of 100 days.

To investigate which factors are associated with the treatment frequency in general, a multifactorial ANOVA was conducted for each age group separately. In Table 5 the geometric means of the 100-day-treatment frequency of the fixed influence factors (region and farm size) for the different age groups are shown. These factors are suggesting effects for region and farm size on the uni-factorial level.

**Table 5:** Geometric means (GM) of the 100-day-treatment frequency of the fixed influence factors for the four different age groups.

	sow			piglet			weaner			fattening pig		
	n	GM	Std	n	GM	Std	n	GM	Std	n	GM	Std
	**region**											
**northwest**	75	0.62	6.7	76	9.87	5.48	72	3.78	6.88	168	2.29	8
**middle**	86	0.76	5.65	71	9.25	5.23	68	4.28	5.87	149	1.6	9.3
**east**	7	3.31	4.1	6	14.1	4.98	6	15.67	9	9	3.4	11.03
**south**	47	0.8	4.03	46	18.07	3.92	41	10.45	2.69	18	2.91	2.21
	**farm size**											
**small**	68	0.65	5.01	56	7.73	6.97	62	2.96	4.07	115	0.93	12.71
**medium**	63	0.63	5.85	60	13.65	3.86	60	4.55	6.8	113	2.24	5.53
**large**	84	0.96	6.01	83	12.5	4.65	65	9.93	5.59	116	3.85	5.7

Following the ANOVA-multi-factorial approach:

neither “region” nor “farm size” had a significant impact on the treatment frequency; except for weaners, where these two fixed factors seemed to have a slight influence (Table 6). Furthermore, it can be noted that the “veterinarian” as well as the interaction between “veterinarian” and “farm size” (except for sows) showed a statistically significant association on the 5%-level. This may indicate the direct relationship of farmers and veterinarians, who specialize in pig farming. Using this model, in general a substantial amount of the variance of the treatment frequency for sows could be explained at 33%, for piglets at 50%, for weaners at 45% and for fattening pigs at 27%.

**Table 6:** Final ANOVA for describing treatment frequencies (target variable: logarithmized 100-day- treatment frequency).

	**sow** (R-Square: 0.326399)				
**source**	**DF**	**Sum of Squares**	**Mean Square**		
**Error**	178	81.6707230	0.4588243		
**Correction Total**	214	121.2449435			
	**DF**	**Type III SS**	**Mean Square**	**F Value**	**Pr > F**
**region**	2	1.34617447	0.67308724	2.64	0.1858
**farm size**	2	0.07275842	0.03637921	0.06	0.9459
**veterinarian**	13	19.42976993	1.49459769	3.26	0.0002
farm size*veterinarian	14	9.12067360	0.65147669	1.42	0.1478
region*veterinarian	4	1.01988934	0.25497233	0.56	0.6951

	**piglet** (R-Square: 0.497702)				
**source**	**DF**	**Sum of Squares**	**Mean Square**		
**Error**	163	49.26920456	0.30226506		
**Correction Total**	198	98.08760267			
	**DF**	**Type III SS**	**Mean Square**	**F Value**	**Pr > F**
**region**	2	0.97108955	0.48554477	1.36	0.3793
**farm size**	2	2.72531103	1.36265551	1.46	0.2682
**veterinarian**	13	33.55440307	2.58110793	8.54	<.0001
farm size*veterinarian	13	12.14350519	0.93411578	3.09	0.0004
region*veterinarian	3	1.06889828	0.35629943	1.18	0.3196

	**weaner** (R-Square: 0.453022)				
**source**	**DF**	**Sum of Squares**	**Mean Square**		
**Error**	149	59.2655024	0.3977550		
**Correction Total**	186	108.3508443			
	**DF**	**Type III SS**	**Mean Square**	**F Value**	**Pr > F**
**region**	2	1.16987186	0.58493593	7.69	0.0426
**farm size**	2	5.76430524	2.88215262	3,96	0.0415
**veterinarian**	13	18.64776524	1.43444348	3.61	<.0001
farm size*veterinarian	15	10.90852450	0.72723497	1.83	0.0355
region*veterinarian	4	0.30418793	0.07604698	0.19	0.9427

	**fattening pig** (R-Square: 0.265086)				
**source**	**DF**	**Sum of Squares**	**Mean Square**		
**Error**	298	212.2190436	0.7121444		
**Correction Total**	343	288.7672195			
	**DF**	**Type III SS**	**Mean Square**	**F Value**	**Pr > F**
**region**	2	0.68633442	0.34316721	0.92	0.4557
**farm size**	2	2.83907983	1.41953991	1.11	0.3513
**veterinarian**	17	23.66596891	1.39211582	1.95	0.0139
farm size*veterinarian	18	23.03261344	1.27958964	1.80	0.0251
region*veterinarian	5	1.85830095	0.37166019	0.52	0.7597

## Discussion

### 1. General study design

Data presented here was created in the setting of a cross-sectional study taken into account the variability in German farming practice. Therefore a stratified approach was used with four regions of different agricultural areas. The formal study base used was the official forms of recording the use of pharmaceutical drugs which are obligatory by law in Germany. Therefore all calculations were based on individual application data on farm level, including the drug, the amount, the number of days, the number of animals treated, and the animal age group respectively on an individual level of treatment. This concept is in contrast to other (monitoring) studies, where mostly the entire amount per farm or even more general groups was recorded and numbers of animals treated and other variables of interest have to be estimated from this aggregated information (see e.g. studies in Denmark [6] or the Netherlands [7]). This investigation was conducted as a follow-up of the study implemented by Merle et al. [13], where the general feasibility of the concept was proofed. The study was set up as a cross-sectional study with representative sampling using recorded information from veterinarians and farmers on the application of antibiotics for the year 2011.

As sales data do not contain any information on the usage of the drugs, it is not possible to combine sales data with real antibiotic application for animals. Bondt et al. [19] and Chauvin et al. [20] also stated that this is inadequate. Therefore, Bondt et al. [19] propose collecting detailed data to enable an assessment on the real antimicrobial usage. In our study this was implemented by using the detailed and informative data of the treatment and delivery forms. These documents allow an evaluation of antimicrobial usage on the level of individual treatment. Furthermore, they allow a categorization of treatments by the individual age groups, which was also considered by Bondt et al. [19]. Thus, on the basis of the used data, we were able to evaluate the consumed quantities and the treatment frequencies for piglets and sows separately from each other. This is a denotative benefit compared to the evaluation of other monitoring programs [6,7], in which sows and piglets are considered as one unit.

### 2. Study population

This study is based upon voluntary participation of farmers and veterinarians. It has to be taken into account that especially those veterinarians and farmers may be open to participation, who usually care about a diligent and thoughtful handling of drugs and who are seeking to improve their management. Thus, it is possible that the real use of antimicrobial substances is higher and a selection bias cannot be completely ruled out. To ensure compliance with the study, all information was pseudo-anonymized and no information was transferred to veterinary authorities. This resulted in a study base of farms, which is closely connected to the figures known from the demographic structures in pig-holdings farms in Germany concerning the regional distribution as well as the structures of farming sizes and general facilities. Therefore, the coverage of livestock places in the study population compared to the total livestock population in Germany is sufficiently high. These figures were evaluated on the basis of the annual German livestock microcensus (Federal Statistical Office, microcensus 2011). In addition these results show a huge variety of treatment frequencies per farm with some extended results (e.g. a farm with a treatment frequency within the 42 day flat-deck period of approximately 75, i.e. a treatment with two active ingredients per day for all animals). This should be seen as an argument that a severe selection bias is not present in the data.

### 3. General measurement of antibiotics use

For evaluating and describing the antimicrobial usage, various definitions and variables have been introduced over time [17,21,22]. Until now, no standardized variables have been established which enable an assessment of the antimicrobial use between different countries. This is basically due to an enormous spread of study designs and data structures linked to different structures in different countries. But, the examination of used quantities on its own allows a general overview of the situation, which is performed by the ESVAC project [5,23,24].

However, active ingredients, which were applied at a high dosage over several days, carry higher weight than those which were applied at a lower dosage. In addition, the frequency of application may be different, depending on the type of drug used and whether they maintain the effect of the active ingredient for a longer time. This may lead to an overvaluation or undervaluation of the situation [25]. Therefore, a comparison between the consumed quantities per active ingredient class and the number of treatment units allows a more differentiated view on the importance of the individual active ingredient classes.

Using an appropriate variable is essential for interpreting the antibiotic usage [26]. In addition, terms were already defined by various authors, which link the used quantity to a specific time period or a definite population. For example, Callens et al. [26] and Vieira et al. [27] apply the so-called “treatment incidence” for describing the antimicrobial use in detail and for comparing the antimicrobial use between farms in a standardized way. For the same reason, Dunlop et al. [28] use the "treatment rate", and van der Fels-Klerx et al. [29] use the NDD (number of daily dosages per average pig year). Although all these terms have the same background, namely the representation of the actual treatments and with it the actual situation, all terms are defined and calculated slightly differently and thus are only conditionally comparable with each other. Therefore, a standardization is necessary [25].

In the context of the analysis presented here, the antibiotic use was examined regarding the used quantities as well as the frequency of treatments. To do this, the variables "treatment unit" and especially "treatment frequency" were used. This term specifies the average number of days an animal of a defined population is treated with one active ingredient within a certain period of time. The treatment frequency does not distinguish between dosages, but it merely indicates how often an animal is treated with one active ingredient. Therefore, low and high dosed active ingredients, as well as treatment of young or elder animals, are equally weighted, unlike pure consumption quantities or terms which are based on dosages and standardized body weights (Jensen et al. [22]). On that account, the treatment frequency is only limited comparable with the DDDA_kg_, which is established in Denmark [6], or the DDD_kg_, which is established in the Netherlands [7].

Whereas the concept in general is comparable to the nDDDA (number of defined daily doses animal; see ESVAC [5]), the main difference is that our calculation is based on detailed knowledge of the number of used applications. In contrast, the number of DDDAs proposed by ESVAC is calculated on the basis of amounts used and dosages assumed for standard weighted animals used, which therefore may be interpreted as an estimator of the treatment frequency used here.

The treatment frequency is comparable between years and regions, and may also refer to an arbitrary population [13]. For calculating the treatment frequency, detailed information about the individual application as well as the population size is needed. Furthermore, if appropriate data are collected, the treatment frequency can also be calculated for each active ingredient separately. This was done by Vieira et al. [27] in a similar manner by using ingredient-related treatment incidence rates.

### 4. Amounts of antimicrobial drug consumption and sales data

In general, among all age groups in pigs the highest amounts of antimicrobials used were tetracyclines, followed by beta-lactams and trimethoprim-sulfonamides. Also in regard to the sales data for all animals species in Germany, tetracyclines and beta-lactams are in first and second place, followed by trimethoprim-sulfonamides, macrolides and polypeptides [23,30].

In the feasibility study of the years 2007/2008, Merle et al. [31] also asserted that tetracyclines were quantitatively most commonly used on pig farms in Germany. This active ingredient class was followed by trimethoprim-sulfonamides and macrolides. Beta-lactams, which were in second place regarding consumed quantities in our study, only ranked fourth in the feasibility study.

The finding of our recent study in general is in line with sales data for 2011 in Germany, where penicillins were the antimicrobial class in second place as regards the sold amount. The trend towards increasing use of beta-lactams and decreasing use of tetracyclines was already discussed by Merle et al. [31]. Falling prices for beta-lactam antibiotics, as well as the compensation for the loss of growth promoters were mentioned as reasons there.

In 2011 sales data on antimicrobial substances used in veterinary medicine in Germany were collected obligatory for the first time. These data based on the announcements of pharmaceutical companies and wholesales, who sold drugs to veterinarians in Germany and were published in context of the third ESVAC report [23,24], in which 25 European countries are compared.

But, sales data is a total on suggested use in all animal species, which prohibited a direct comparison for the data presented here for pigs only. In a recent publication we compared the distribution of active ingredient classes for these sales data with the consumption data of our study for all animal species under study, which are closely related. Taken into account that data on turkey and other species was not available, an extrapolation from study data to sales data was sufficient [32].

Jordan et al. [33] for Australia and Rajić et al. [34] for Canada asserted that the used amounts of beta-lactams, tetracyclines and sulfonamides were the highest.

It is important to note that these are active ingredient classes, which are highly important (tetracyclines and sulfonamides) or even critically important (beta-lactams) for human medicine [35]. However, Morley et al. [36] defined exactly these active ingredient classes as antibiotics assigned to the “Primary Use Category” in veterinary medicine. It is constantly discussed, that the antibiotic use in food-producing animals might have a negative influence on human health [2]. Nonetheless, Aarestrup et al. [37] cautioned about an increased risk to human health if the antimicrobial consumption changes towards more critically important antimicrobials as a result of reduction measures. However, the broad spectrum and their relatively low costs may be a reason for the high consumption quantities of these active ingredient classes. Furthermore, these substances mostly require high dosages, which lead to an associated high consumption quantity.

Some authors also report, that these active ingredient classes were often used for metaphylactic matters [38]. But for Germany this could not be improved by the means of our study design, which based on official forms only. Here, metaphylactic use was not reported as an indication due to the general rules stated in the guidelines on the prudent use of antibiotics in veterinary medicine [1].

In sows, tetracyclin, trimethoprim-sulfonamides and amoxicillin were mostly used in this study. In piglets, amoxicillin, tetracyclin, trimethoprim-sulfonamides and colistin were mostly used.

Dunlop et al. [28] show that especially gentamicin and penicillin were used in piglets, and that in lactating sows especially penicillin was used. Beta-lactam antibiotics also play an important role in this study. Jensen et al. [39] reported that beta-lactams seem to have the highest importance in the treatment of piglets and sows over a period of time (2002–2008).

According to Dunlop et al. [28], tetracyclin and tylosin were only used to a very small extent in piglets, but these substances were often used in fattening pigs [40]. In the meantime, these substances seem to have gained in importance for treating piglets. According to Dunlop et al. [28], tetracyclin was the second most used active ingredient in sows.

Also, in the study of Jensen et al. [39], tetracyclines were the fourth most used active ingredient in piglets and sows. Therefore, tetracyclines still have a quite high priority in the treatment of piglets and sows.

Jensen et al. [39] reported that macrolide antibiotics, which implies the active ingredient tylosin, have lost importance in the treatment of piglets and sows and have been replaced by pleuromutilins. This could not be confirmed for piglets in our study; here, pleuromutilins only played a minor role. Nevertheless, in sows, the amounts of used pleuromutilins and macrolides were nearly the same.

Similar to this study, Dunlop et al. [28] and Jensen et al. [39] found out that potentiated sulfonamides are used extensively for treating piglets and sows. As well as tetracyclines, potentiated sulfonamides also seem to have a quite high priority in the treatment of piglets and sows.

In weaners, amoxicillin, tetracyclin, chlorotertacyclin as well as trimethoprim-sulfonamides and colistin in almost equal quantities, were mostly applied in this study. In fattening pigs the active ingredients amoxicillin, tylosin, chlorotetracyclin and tetracyclin were mostly used.

According to Dunlop et al. [28], already over a decade ago weaners and fattening pigs were mostly treated with beta-lactams (here penicillin). In Australia, beta-lactams, tetracyclines and sulfonamides are most commonly used [33]. In Spain and Belgium, macrolides, tetracyclines, beta-lactams and polypeptides are mainly used in weaners and fattening pigs [38,41,42].

According to Callens et al. [26] the most commonly used active ingredient classes such as potentiated sulfonamides and tetracyclines have remained the same in quantity over the years, but the importance of individual active ingredient classes has shifted. Thus, modern antimicrobials (e.g. cephalosporins) are increasingly used, because they promise advantages to the farmer relating to their long and potent acting [26]. In addition, these substances have to be administered in lower doses and less frequently, whereby they are of no importance regarding quantities used. To be able to evaluate the importance concerning the actual use of these substances, and the shift from the use of substances of low importance in human medicine to substances of high importance for medicine, it is essential to collect detailed data on each antimicrobial used and to describe the individual usage patterns by use of appropriate terms [21,25,26].

98% of the used quantity was administered orally, whereas in this connection especially treatments of weaners and fattening pigs led to this distribution. This observation corresponds with the in ESVAC reported high amounts of oral powder of antibiotic substances [23]. Merle et al. [13] also reported that 97% of the amount used in pigs was administered orally. Also Timmerman et al. [38] showed this distribution between oral and parenteral administered quantities in pigs, where group treatments were also conducted for reasons of metaphylaxis especially in weaners and fattening pigs. In this context, higher quantities of antimicrobial substances had to be used compared to parenteral single treatments because, on the one hand, orally administered substances have to be dispensed at higher doses, and, on the other hand, higher quantities are needed for treating a high number of animals at the same time. This also clearly shows that the consideration of pure quantities may lead to a distorted picture.

### 5. Indication for treatment

Among all age groups, respiratory diseases represent the main indication for the use of antimicrobials. It should be stressed here again that in Germany prophylactic usage of antibiotics is not allowed. The evaluation of reasons for using antimicrobial substances based on the documentation on official forms, on which any treatment administered to the animal is documented obligatory by the veterinarian. Therefore, the documentation and report of misuse, off-label use and prophylactic use is not possible with this study design.

Respiratory diseases are primarily treated with beta-lactam antibiotics in piglets, weaners and fattening pigs, but not in sows. Except for sows, intestinal diseases are the next most frequent indication for antibiotic treatment. In our study, mainly macrolides (tylosin) and polypeptides (colistin) are used for treating intestinal diseases. Chauvin et al. [20] also reported that respiratory and intestinal diseases are the most common indications for antibiotic treatment. Here, colistin and tylosin were also most commonly used against intestinal diseases, but against respiratory diseases mainly tetracyclines were used. Casal et al. [41] also reported that colistin was mainly used for treating intestinal disease, even with a high prophylactic use, i.e. without defined diagnosis. Casal et al. [41] also reported that respiratory diseases were also treated mainly with tetracyclines, with beta-lactams being only the second most used active ingredient and that prophylactic treatments play an important role again. Jensen et al. [39] commented that intestinal diseases constitute the main indication, which is treated with macrolides and tetracyclines to a large extent. However, especially in fattening pigs, also pleuromutilins are used in one third for that indication. In this study, respiratory diseases are listed as the second most common indication, especially tetracyclines playing the main role in their treatment, beta-lactams making up only a small percentage. In another study, intestinal diseases also seem to represent the main indication [38]. The increase in prescriptions against intestinal diseases and the associated decrease of prescriptions of drugs against respiratory diseases was reported by Arnold et al. [25]. In contrast, Jensen et al. [39] reported rather an increase in the treatment of respiratory diseases, and a decrease in the treatment of intestinal diseases.

The second most common indication for sows is diseases of the reproductive organs, which are primarily treated with tetracyclines. According to Chauvin et al. [20], tetracyclines were rarely used for treatment of reproductive organ diseases. Here, in particular sulfonamides, fluoroquinolones and beta-lactams played a role. A similar picture is also described by Jensen et al. [39].

Considering seasonal effects in view of antimicrobial consumption might give an allusion to seasonal dependencies on disease patterns. However, it must be emphasized that different consumption levels by season may be caused by differences in the number of animals kept in the individual time periods. This could not be answered with our data due to the documentation of average numbers of animals kept over one year only.

Thus, the highest drug use in piglets was detected in autumn in our study. In autumn, amoxicillin was used in highest quantities. Furthermore, amoxicillin was used for treating respiratory diseases, which was the main indication for treating piglets in our study. Based on these results, it could be speculated that there was an increase in respiratory diseases due to falling temperatures in autumn and the associated higher susceptibility to infections in younger animals. Nevertheless, this could not be confirmed for weaners and fattening pigs; there the hightest amount of antimicrobials usually consumed for treating respiratory diseases was in winter and spring. In contrast to theses observations, recently, Sanchez-Vazquez et al. [43] described that pulmonal lesions were observed most frequently in slaughtered pigs during November and December.

As regards colistin and tylosin, the antimicrobials commonly used for treating intestinal disorders, again there was no consistent picture. Low amounts were used in winter for piglets and fattening pigs, but not for weaners. As regards weaners and fattening pigs, quite high amounts were used in spring to treat dysenteriae.

When looking at sows, the highest drug use in our study was detected in spring, with the highest amounts of tetracycline used in spring. In addition, tetracycline is mainly used for treating respiratory diseases and diseases of the reproductive organs, which were at the top of the indications in sows in our study. However, sows are continuously used throughout the year for producing piglets, so that a seasonal accumulation of diseases of the reproductive organs could be ruled out.

In the feasibility study, no seasonal effects were observed with regard to used quantities in all age groups covered in the study [44], but looking at antimicrobial consumption in pigs in a certain rural district, more than half of the amounts were used in summer [45]. In the other seasons only one quarter of this amount was consumed. However, the number of treated animals was the highest here in summer, in fact even more than twice as high than in other seasons.

### 6. Frequency of antimicrobial drug use

In our study, the highest median treatment frequency—for a standardized period of 100 days—was calculated for piglets with 14.7 days, followed by weaners with 6.6 days, and fattening pigs with 3.7 days. The lowest rate was calculated for sows with median 0.9 days per 100 days. In the previous feasibility study, arithmetic means had been reported. There, the treatment frequency for piglets was lower with 6.1 days per 100 days, but at the same magnitude for sows with a treatment frequency of 0.9 days per 100 days. For fattening pigs, the average treatment frequency, which was observed in the feasibility study, was higher with 4.6 days per 100 days on average [31] compared to this study. In an antibiotic usage monitoring study, which was undertaken in Lower Saxony, a region in the North of Germany, in 2011, a treatment frequency of 4.6 days within the period of a fattening passage (115 days) for fattening pigs was calculated on average [46]. Converted to 100 days, this would result in a treatment frequency of 4.0 days and thus in the same order of magnitude.

In summary, while the treatment frequencies of sows and fattening pigs calculated in these three studies are similar to each other, the treatment frequency of piglets is more than twice as high in our study than in the feasibility study. This could be explained by a misclassification of weaners and piglets, which may have had impact on the results, because data is from veterinary practitioners, who were note standardized in beforehand. In this case, treatment frequency for piglets may have been overestimated and the treatment frequency of piglets may have been less than the calculated value, while the actual treatment frequency of weaners was possibly much higher than the calculated median value.

Merle et al. [31] already compared the results of the feasibility study with those of other European member states with a more or less similar farming structure. As described above, most consumption data based on amounts and uses theoretical DDDA for estimating the antibiotics usage. Our data based on UDDA, a fact that does not allow a direct comparison. Merle et al. [47] constituted the differences between UDD and ADD in detail. However, most important is that nUDDA uses the number of treated animals as well as the applied dosage directly without any estimation. This may reduce the variance of the given information. In addition, veterinarians choice of dosage is linked to the situation indicated within the farm, which avoid bias due to the accounting via DDDA. This has to be taken into account comparing our data with results of other studies.

Bos et al. [24] calculated the ADDD/year for fattening pigs and sow—piglet- units in the Netherlands for 2011. For fattening pigs a median ADDD/year of 3.0 was reported, which implies 3 days of treatment per fattening pig within one year in the Netherlands. Thus, the actual treatment frequency of fattening pigs in Germany is higher than the estimated treatment frequency in the Netherlands. For sow-piglet-units a median ADDD/year of 9.3 was calculated. This is difficult to compare to our detailed results for piglets and sows, but the Dutch estimated treatment frequency of sows and piglets also seem to be lower than the German actual treatment frequencies for sows and piglets.

Looking at the treatment frequencies concerning individual active ingredient classes, beta-lactams represent the active ingredient class that was used on most farms among all age groups, with a treatment frequency of 4.3 days for piglets, 0.2 days for sows, 2.2 days for weaners and 0.8 days for fattening pigs, respectively. In contrast, Vieira et al. [27] noted the highest treatment incidence rates within 100 pig-days under risk for tetracyclines (0.7 ADD/100 pig-days under risk) and macrolides (0.44 ADD/100 pig-days under risk) for fattening pigs in Denmark. Bos et al. [24] calculated an ADDD/year per active ingredient class and animal group for farms in the Netherlands. Here, also for tetracyclines the highest values were evaluated both in fattening pigs and in sow—piglet—units. In second place and third place beta-lactams and trimethoprim-sulfonamides were shown for Dutch fattening pigs.

However, in the feasibility study Merle et al. [13] determined that looking at treatment frequencies concerning individual active ingredient classes, trimethoprim-sulfonamides is by far the most commonly used in piglets, sows and fattening pigs. In the feasibility study, in piglets trimethoprim-sulfonamides was applied 9.6 days (arithmetic mean) per 100 days, in sows 0.3 days (median) per 100 days and in fattening pigs 3.8 days (arithmetic mean) per 100 days, respectively. In our study, trimethoprim-sulfonamides was only used in 36% of all sow keeping farms, in 17% of all piglet keeping farms and in 14% of all farms keeping fattening pigs. Within these farms, trimethoprim-sulfonamides was applied to piglets at 6.4 days, to sows at 0.3 days and to fattening pigs at about two days over a period of 100 days.

As another example, on all farms which used polypeptides for treating infections, piglets were treated 9.7 days (median) per 100 days with polypeptides. Here, especially colistin plays the major role. Polypeptides are used for the treatment of respiratory infections as well as intestinal infections [48], which constitutes the two most common reasons for treating piglets. Chauvin et al. [20] reported that polypeptides are used almost exclusively against intestinal diseases. Timmerman et al. [38] calculated a treatment incidence for colistin in fattening pigs (TI_UDDpig_: 32.8), which is in third place compared to other active substances. The fact that the treatment incidence is not directly identical with the treatment frequency and that other authors [27,39] do not report information on polypeptides, leads to difficulties in a direct comparison.

As polypeptides are quite lowly dosed compared to sulfonamides and tetracyclines, which are mainly used against intestinal and respiratory diseases, according to Jensen et al. [39], polypeptides only play a minor role regarding quantities used. Therefore, the actual importance of this substance is shown by looking at the individual treatments only. Once again, this underlines the advantage of treatment frequency.

Vieira et al. [27] noted that there is a significantly higher treatment frequency on small farms keeping fattening pigs than on larger farms. This was justified by the higher entry of pathogens based on worse hygiene management on smaller farms (animal keeping as a hobby). In contrast, van der Fels-Klerx et al. [29] determined that the treatment frequencies on large farms keeping sows and fattening pigs is higher than on smaller farms. This is explained with a higher level of exposure of animals to infective agents on large farms. In our study the influence of the factors "farm size", "region" and "veterinarian", as well as their interaction, on the treatment frequency were studied with an analysis of variance model. Here, the farm size on its own seemed to have no influence on the treatment frequency, except a slight influence on the treatment frequency of weaners.

Considering the treatment frequency within the age groups it becomes obvious that piglets are far the most commonly treated age group, followed by weaners and fattening pigs. In contrast, sows are treated rarely. In addition, the model has shown that in this study the farm size only had a significant influence on the treatment frequency in interaction with the veterinarian. This could be justified due to the fact that several veterinarians advise farms of different sizes and that they follow different treatment strategies. Furthermore, the veterinarian alone has a significant impact on the treatment frequency. This may also be justified with different specializations of the different veterinarians. Thus, some veterinarians are specialized in the supervision of fattening pigs, whereas others are specialized in the supervision of sows and piglets, whereby here also different treatment strategies are followed.

Callens et al. [26] also observed that 90% of the treatments take place during the first 10 weeks of life, whereas fattening pigs are treated rarely. These results differ from those of Dunlop et al. [28], who determined that most of the individual treatments were given to piglets and sows at parturition, fattening pigs being treated scarcely and weaners being treated very rarely. Here, the low frequencies of treatment in weaners and fattening pigs are explained by good farm and hygiene management of the corresponding farms. Jensen et al. [39] determined that younger animals are treated more often than older animals (weaners more often than fattening pigs). Here, the frequency of treatment in piglets and sows cannot be interpreted because they were not considered individually, but as a unit. A higher treatment frequency of younger animals may be due to a higher susceptibility to infection. Especially on farms keeping weaners, which include animals from different origins as well as on farms with a high animal movement, a high infection pressure and the resulting greater need for treatment could exist [29,42].

The invariably right-skewed distribution of the treatment frequencies shows for all age groups the majority of farms having rather low and only a few farms having higher treatment frequencies. This observation is also documented by Bos et al. [24] for the antibiotic usage on farm level in the Netherlands. Jordan et al. [33] observed a distribution with normally distributed antimicrobial use indices for pig farms in Australia. Here, a moderate antimicrobial use index was determined for the majority of farms, but there were a few farms with low or high antimicrobial use indices in equal shares.

## Conclusion

In Germany a representative scientific collection of data on the antibiotic use in food-producing animals is feasible. The legally binding requirement to document all treatments and deliveries provides an excellent data source, allowing for a penetrative analysis at an individual animal level. Therefore, conclusions about the actual usage of antibiotics are allowed, both on the level on amounts of drugs as well as on the level of number of used daily doses (per antibiotic class).

In total amounts, tetracyclines, beta-lactams and trimethoprim-sulfonamides are the most commonly used active ingredient classes in pigs in Germany. This general observation does not differ from the results of other antibiotic consumption studies in other countries. However, if data is stratified in the different age-classes of pig's life, the using pattern changes and beta-lactams, macrolides and also polypeptides seem to have become more important in treating pigs in Germany.

In the VetCAb study, the median treatment frequency—for a standardized period of 100 days—was calculated for piglets with 14.7 days. Weaners are treated at 6.6 days, and fattening pigs at 3.7 days. The lowest rate was calculated for sows with median 0.9 days per 100 days. These treatment frequencies only differ little from the treatment frequencies calculated in earlier antibiotic consumption studies, which were carried out in Germany.

Most consumption data based on amounts and uses theoretical DDDA for estimating the antibiotics usage. The data used in the VetCAb study based on UDDA, a fact that does not allow a direct comparison. However, most important is that nUDDA uses the number of treated animals as well as the applied dosage directly without any estimation. This may reduce the variance of the given information. Thus, actual treatment frequencies possibly differ from estimated treatment frequencies.

The data shows, that same antimicrobial substances both in human and veterinary medicine are used, which may cause a development and a transfer of resistance. But, linking the data collected in our study with human health is not allowed due to a lack of similar information for human treatment.

Our study is the first cross-sectional study on antimicrobial usage in pig farming in entire Germany. As a first reference point our data may be used to develop rules for regular or elevated use of antibiotic substances of German farming practice to describe the situation. However, without accompanying studies on the resistance level in livestock in Germany, it remains difficult to realize comparative observations on the two research sectors.
